# Proton-pump inhibitors are associated with increased risk of prosthetic joint infection in patients with total hip arthroplasty: a case-cohort study

**DOI:** 10.1080/17453674.2021.1920687

**Published:** 2021-05-12

**Authors:** Maarten M Bruin, Ruud L M Deijkers, Roos Bazuin, Erika P M Elzakker, Bart G Pijls

**Affiliations:** a Department of Orthopedic Surgery, HagaZiekenhuis, Den Haag; b Department of Medical Microbiology, Hagaziekenhuis, Den Haag; c Department of Orthopedic Surgery, LUMC, Leiden, The Netherlands

## Abstract

Background and purpose — Proton-pump inhibitors (PPI) have previously been associated with an increased risk of infections such as community-acquired pneumonia, gastrointestinal infections and central nervous system infection. Therefore, we evaluated a possible association between proton-pump inhibitor use and prosthetic joint infection (PJI) in patients with total hip arthroplasty (THA), because they can be stopped perioperatively or switched to a less harmful alternative.

Patients and methods — A cohort of 5,512 primary THAs provided the base for a case-cohort design; cases were identified as patients with early-onset PJI. A weighted Cox proportional hazard regression model was used for the study design and to adjust for potential confounders.

Results — There were 75 patients diagnosed with PJI of whom 32 (43%) used PPIs perioperatively compared with 75 PPI users (25%) in the control group of 302 patients. The risk of PJI was 2.4 times higher (95% CI 1.4–4.0) for patients using PPI. This effect remained after correction for possible confounders.

Interpretation — The use of PPIs was associated with an increased risk of developing PJI after THA. Hence, the use of a PPI appears to be a modifiable risk factor for PJI.

One important aspect of the prevention of prosthetic joint infection (PJI) is preoperative optimization of modifiable risk factors (Kunutsor et al. 2016). Medication may be an important category of modifiable risk factors, since they can be temporarily discontinued perioperatively or they can be switched to a less harmful alternative. Proton-pump inhibitors (PPI) are of special interest, because they have been associated with an increased risk of infections such as community-acquired pneumonia, gastrointestinal infections, and central nervous system infections (Lambert et al. 2015, Cunningham et al. 2018, Hung et al. 2018). The increased risk of these infections is probably due to the fact that PPIs decrease the effectiveness of neutrophils (Aybay et al. 1995, Agastya et al. 2000, Zedtwitz-Liebenstein et al. 2002). This increased risk of infection may also apply to total hip arthroplasty (THA), possibly leading to increased risk of PJI. However, the effect of PPIs on the risk of PJI is currently unknown. Therefore, we evaluated a possible association between perioperative PPI use and early-onset prosthetic joint infection in patients with total hip arthroplasty.

## Patients and methods

This is a case-cohort study. We collected data of patients treated with THA between January 2009 and December 2017 in HAGA hospital in the Netherlands, which is a high-volume teaching hospital. A case-cohort design was chosen to allow efficient assessment of the risk factors (PPI use and confounders) and we used the approach of Cai and Zeng (2004) for sample size considerations. This design provides similar effect estimates and standard errors compared with full cohorts, while at the same time allowing for a high level of detail. Comparable studies evaluating risk factors for PJI with similar sample size were able to detect risk factors (Choong et al. 2007, Dowsey and Choong 2008).

### Base-cohort and controls

5,512 patients with a primary THA were identified. We excluded patients with hemi-arthroplasty, revision surgery, and THA through an approach other than the direct anterior approach (DAA). All patients received as perioperative prophylaxis either cefazolin for low-risk patients or vancomycin and ciprofloxacin for high-risk patients as per hospital protocol. For every case, we randomly selected 4 controls from the base cohort using a random-number generator. This resulted in the study population of 75 cases and 302 controls. 3 cases were also included as controls, which is normal in case-cohort designs. This phenomenon indicates that the selection of controls was truly random: at baseline (immediately after THA) PJI is not yet diagnosed and some patients will develop PJI postoperatively. Therefore, in a random sample of controls at baseline, the percentage controls who develop PJI should be similar to the incidence of PJI in the whole cohort (Prentice 1986). This was the case in our study: 1.4% (72 of 5,512) is similar to 1% (3 of 302).

### Cases

The cases comprise patients with early-onset PJI. Early-onset PJI was defined as PJI occurring within the first 3 months after surgery (Tande and Patel 2014). The diagnosis of PJI was made according to the major and minor MSIS criteria (Parvizi et al. 2011).

To ensure that we included all cases we consulted the Dutch Arthroplasty Register (LROI) to check whether revision for infections or DAIRs (debridement, antibiotics, and implant retention) had been done in other hospitals for patients in the study cohort (van Steenbergen et al. 2015). The Dutch Arthroplasty Register identified no revisions for infections or DAIRs that had been done in other hospitals that we were unaware of for patients in our cohort.

### Data collection and statistics

Data was extracted from the hospital information system HiX (https://chipsoft.com/solutions/532/HiX-the-most-innovative-HIS-EHR) or paper medical records by the researchers and was collected in Castor Electronic Data Capture (https://www.castoredc.com/clinical-data-management-system/). For all patients the demographic data, perioperative use of PPIs, and potential confounders such as vitamin K antagonist use was collected. Preoperative medication use was recorded by anesthetists as part of routine preoperative screening.

PJI is a time-to-event outcome and effect of PPI use on PJI risk was analyzed with Kaplan–Meier statistics and weighted Cox proportional hazards regression. We used a weighted method according to Barlow et al. (1999) to calculate the hazard ratios (HR) and their 95% confidence interval (CI) (Barlow et al. 1999). Sub-cohort controls are weighted by the inverse of the sampling fraction α (= 302 controls/5,512 entire cohort = 0.055) and the case weight outside the sub-cohort is always 1 at failure. The following weights were thus applied: 1 for a case outside the sub-cohort at failure, 18 (= 1/0.055) for a case in the sub-cohort before failure, 1 for a case in the sub-cohort at failure, 18 for a sub-cohort control. A Kaplan–Meier curve was plotted to ensure that the proportional hazard assumption was not violated.

We selected confounders based on the following criteria (Rothman et al. 2008):

1. A confounding factor must be an extraneous risk factor for the disease (i.e., PJI).
2. A confounding factor must be associated with the exposure (i.e., PPI) under study in the source population.
3. A confounding factor must not be affected by the exposure or the disease. In particular it cannot be an intermediate (mediator) step in the causal path between exposure and the disease.

Demographic factors such as age, sex, and BMI have been associated with PJI as well as PPI use, so they were considered possible confounders (Pedersen et al. 2010, Hálfdánarson et al. 2018, Antonelli and Chen 2019).

Regarding criterion 2, PPIs are prescribed when using NSAIDs, acetylsalicylic acid, certain immunosuppressive drugs, vitamin K antagonists and polypharmacy in some patients (see https://www.farmacotherapeutischkompas.nl/bladeren/ indicatieteksten/maagbescherming). Regarding criterion 1, anticoagulants, immunosuppressive drugs, and polypharmacy have been shown to be risk factors for PJI and do not violate criterion 3, so they were considered possible confounders and included in the model (Pedersen et al. 2010, Antonelli and Chen 2019). NSAIDs have been shown not to be associated with PJI and were therefore not considered a possible confounder (Pedersen et al. 2010). Taken together the following factors met the criteria above and were thus included in the model as possible confounders: age, sex, BMI, acetylsalicylic acid use, use of vitamin K antagonists, immunosuppressive drug use, and polypharmacy. Polypharmacy was defined as the daily use of 5 or more different medications (Masnoon et al. 2017). All analyses were conducted using R package “coxphw” to allow for calculation of robust standard errors (Dunkler et al. 2018).

### Ethics, data sharing, funding and potential conflicts of interest

This case-cohort study was approved by our institutional ethics committee (T17-111) and we comply with the STROBE guidelines for reporting. The data is available upon reasonable request by contacting the corresponding author. The authors received no financial support for the research, and declare no conflict of interests.

## Results

There were 75 cases of PJI in 5,512 primary THAs, resulting in an infection rate of 1.4%. The causative micro-organisms were: *S. aureus* (n = 32), coagulase-negative staphylococci (n = 23), *P. aeruginosa* (n = 8), *E. faecalis* (n = 13), *E. faecium* (n = 1), *Enterobacteriaceae* (n = 23), streptococci (n = 6), and *Corynebacterium ssp* (n = 4); the numbers add up to more than 75 cases, because the infection was polymicrobial in 28 hips. The majority of cases were early-onset postoperative: 73 (of 75) cases were treated with a DAIR within 3 months after THA. There were 2 late (acute hematogenous) cases with onset of symptoms less than 4 weeks prior to DAIR procedure. For 74 cases, 2 or more perioperative cultures were positive. The remaining case had 1 positive perioperative culture and the minor MSIS criteria were taken into account. The mean duration between arthroplasty and DAIR was 36 days (SD 90 days). The mean follow-up for the controls was 3.8 years (SD 2.3 years; range 15 to 3,361 days) (Table 1).

**Table 1.** Patient demographics. Values are count (%) unless otherwise specified

	Cases	Controls
Variable	n = 75	n = 302
Age, mean years (SD)	69 (10)	68 (11)
Female sex	42 (56)	188 (62)
BMI, mean (SD)	30 (5.4)	27 (4.2)
Obesity (BMI > 30)	28 (38)	57 (19)
Proton pump inhibitor	32 (43)	75 (25)
Acetylsalicylic acid	17 (23)	46 (15)
Vitamin K antagonist	16 (21)	14 (4.6)
Immunosuppressive drugs	7 (9.3)	12 (4.0)
Polypharmacy **^a^**	37 (49)	109 (36)

Of the 75 patients with PJI, 32 patients (43%) used PPIs perioperatively compared with 75 (25%) in 302 patients in the control group (crude HR 2.4; CI 1.4–4.0; Table 2). After multivariable adjustment, the risk for PJI remained 2 times higher in patients using PPIs perioperatively compared with patients not using PPIs (HR 1.9; CI 0.4–10; Table 3). The Figure shows the risk of PJI according to PPI use (weighted 1-minus-survival Kaplan–Meier plot). In a sensitivity analysis the 2 late acute hematogenous PJI cases were excluded and the results remained similar: HR 2.3 compared with HR 2.4 in the original analysis.

**Figure 1.**
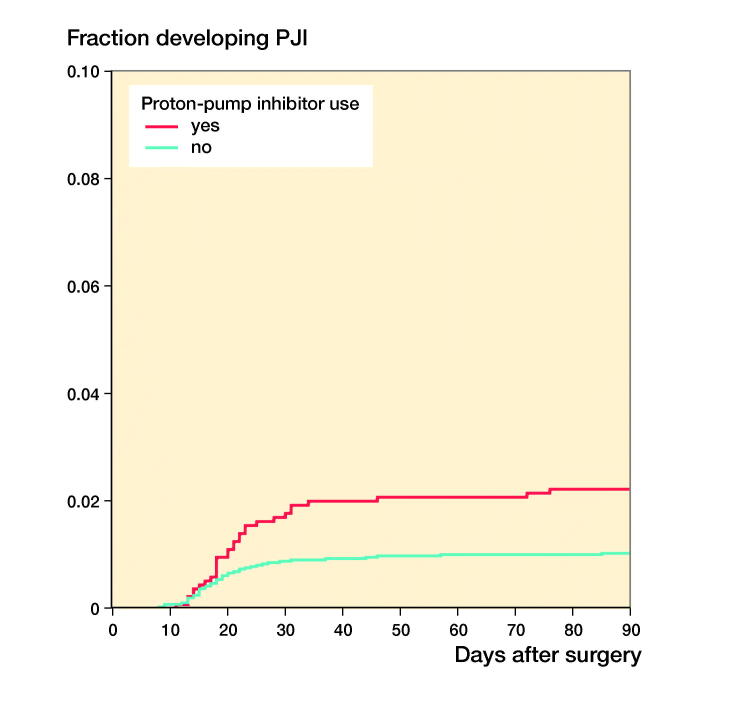
Graph showing risk of prosthetic joint infection (PJI) according to PPI use (weighted 1-minus-survival Kaplan–Meier plot).

**Table 2.** Univariable weighted Cox proportion hazard regression model

Risk factor	HR (95% CI)
Age, years	1.0 (1.0–1.0)
Sex (male)	0.8 (0.5–1.1)
Obesity (BMI > 30)	2.5 (1.6–4.0)
Proton pump inhibitor	2.4 (1.4–4.0)
Acetylsalicylic acid	1.6 (0.9–2.8)
Vitamin K antagonist	5.4 (3.1–9.2)
Immunosuppressive drugs	2.4 (1.1–5.3)
Polypharmacy ^a^	1.7 (1.1–2.7)

**^a^** Defined as the daily use of ≥ 5 different medications.

HR: hazard ratio. CI: confidence interval.

**Table 3.** Multivariable weighted Cox proportion hazard regression model for PPI

Factor	HR (95% CI)
Proton pump inhibitor use	
Crude ^a^	2.4 (1.4–4.0)
Proton pump inhibitor use adjusted for	
Model 1: age	2.3 (1.4–4.0)
Model 2: sex	2.4 (1.3–4.3)
Model 3: BMI	1.9 (1.1–3.3)
Model 4: acetylsalicylic acid use	2.3 (1.2–4.3)
Model 5: vitamin K antagonist use	2.2 (1.0–5.0)
Model 6: immunosuppressive drug use	2.4 (1.1–5.4)
Model 7: polypharmacy.^b^	2.2 (1.1–4.1)
Model 8: all above	1.9 (0.4–10)

**^a^** Crude = HR from univariable model (Table 2).

**^b^** Defined as the daily use of ≥ 5 different medications.

## Discussion

Prosthetic joint infection in total hip arthroplasty is a severe and challenging complication. Therefore, we think preoperative screening for patients with increased risk, optimizing modifiable risk factors before surgery, and counseling patients is important. In this case-cohort study, we found that the use of PPIs is associated with an increased risk of developing PJI after THA.

The incidence of PJI in our base cohort of patients with THA through DAA over a period of 9 years was 1.4%. This is within range of reported infection rates for the DAA from other articles (Aggarwal et al. 2019). We are not aware of other clinical studies describing an association between PPIs and an increased risk of PJI. Also, no clear mechanism for an increase in the incidence of PJI has been described for PPI use. However, several articles describe the impact of PPI use on the immune system. Agastya et al. (2000) reported that PPIs might suppress the innate immune responses by interfering with the functionality of the neutrophils. Also, Liu et al. (2013) found PPIs might inhibit the activity of lysosomal enzymes and alter enzyme functions. Zedtwitz-Liebenstein et al. (2002) designed an experiment where human volunteers received a single dose of omeprazole resulting in decreased bactericidal activity of the neutrophils. The innate immune response and neutrophils have an important role in the host defense response against bacteria. Chronic treatment with PPIs could make patients more susceptible to bacterial infections due to the impaired immune response (Hung et al. 2018).

Malnutrition caused by PPI use may be an alternative mechanism for the observed increased risk of PPIs for PJI. Malnutrition is described as a risk factor for PJI and it is also associated with delayed wound healing, persistent wound drainage, and increased susceptibility to infections (Baek 2014, Pruzansky et al. 2014, Rezapoor and Parvizi 2015). In the study by Kinoshita et al. (2018), PPIs were identified as a possible cause for hypomagnesemia, calcium deficiency, and low vitamin B12. However, it is unclear if and how this effects wound healing and the risk of PJI.

Our study has several limitations. 1st, there is a slight possibility that we have missed early PJI despite our study design and despite consulting the Dutch Arthroplasty Registry, which can be considered non-differential misclassification (Rothman et al. 2008, van Steenbergen et al. 2015, Veltman et al. 2018). In most situations, non-differential misclassification of a binary disease will produce bias towards the null (no effect) (Rothman et al. 2008). This means that if we were to have missed cases (for instance acute hematogenous infections) our estimates for the risk factors would be on the conservative side. Therefore, missing cases would not lead to false identification or overestimation of risk factors for early PJI. 2nd, adjusting for all confounders, model 8 (see Table 3) showed a wider 95% CI and included 1.0; this is limited by the sample size. With an increase in the sample size, the 95% CI would become narrower, while the effect size would stay relatively constant (Lee 2016). 3rd, due to the observational design the observed effect between the use of PPI and the development of PJI should be interpreted as an association, so further research is necessary to determine possible causality (Grimes and Schulz 2002). At present, the potential benefit of temporarily stopping PPIs or switching to another medication group (e.g., histamine blockers or antacids) should be weighed against the risk of PJI on an individual basis.

In conclusion, the results of our case-cohort study showed that the use of PPIs is associated with an increased risk of developing PJI after THA. Hence, the use of PPIs appears to be a modifiable risk factor for PJI.
